# Hip Fractures in Persons with Stroke

**DOI:** 10.1155/2013/954279

**Published:** 2013-04-04

**Authors:** Åsa G. Andersson, Åke Seiger, Peter Appelros

**Affiliations:** ^1^Departments of Geriatrics and Neurology, Örebro University Hospital, 701 85 Örebro, Sweden; ^2^NVS Department, Karolinska Institutet, 171 77 Stockholm, Sweden

## Abstract

*Background*. Our aim was to determine the incidence of hip fractures within two years after stroke, to identify associated factors, to evaluate which test instruments that best could identify people at risk, and to describe the circumstances that prevailed when they sustained their hip fractures. *Method*. A total of 377 persons with first-ever stroke were followed up for a 24-month period. Stroke severity, cognition, and associated medical conditions were registered. The following test instruments were used: National Institutes of Health Stroke Scale, Mini-Mental State Examination, Berg Balance Scale, Timed Up & Go, and Stops Walking When Talking. *Result*. Sixteen of the persons fractured their hip within the study period, which corresponds to an incidence of 32 hip fractures per 1000 person-years. Persons with fractures more often had impaired vision and cognitive impairment and more had had previous fractures. Of the investigated test instruments, Timed Up & Go was the best test to predict fractures. *Conclusion*. The incidence of hip fractures in persons with stroke was high in this study. Persons with previous fractures, and visual and cognitive defects are at the greatest risk. Certain test instruments could be used in order to find people at risk, which should be targeted for fall preventive measures.

## 1. Introduction

Falls are a common consequence of stroke [1]. Hip fractures constitute one of the most serious consequences of falls, since they are a major cause of morbidity and mortality in the elderly [2]. Compared with the general population, stroke survivors have an increased risk of hip fracture within the first year after the stroke [3]. The risk is up to 4 times higher than that for age-matched control subjects [4, 5]. Conversely, among persons with femoral neck fractures, the prevalence of previous stroke ranges from 16.4% to 39.3% [6]. 

Whether a fracture occurs when a person falls largely depends on the type and severity of the fall [7, 8]. As a result of impaired locomotor function, persons with stroke tend to fall towards the weak side, and therefore hip fractures occur more frequently on that side [5, 9]. They may also have a reduced ability to stretch the arm on the weak side in order to soften the fall. Furthermore, it has been suggested that bone resorption occurs rapidly after stroke, which would increase the risk for hip fracture still more [10, 11].

In order to prevent hip fractures, it is important to identify which persons are at the greatest risk and to identify situations in which hip fractures occur. It is also of interest to know if test instruments used in physiotherapeutic practice could identify persons prone to suffer hip fractures.

In the present study our aim was to determine the incidence of hip fracture within two years after stroke. A second aim was to characterize the persons who fractured their hip regarding demographic and medical factors, as well as to evaluate test instruments that are able to predict hip fractures. A further aim was to describe the circumstances that prevailed when the persons sustained their hip fractures.

## 2. Methods

### 2.1. Subjects

The participants in this prospective, longitudinal study are the persons included in a community-based stroke-incidence study. The incidence study was carried out in Örebro during a 12-month period, from February 1, 1999, to January 31, 2000 [12]. Stroke cases were searched inside as well as outside the hospital and identified in several overlapping ways. Persons who fractured their hip within two years after the stroke were identified.

### 2.2. Inclusion and Exclusion Criteria

The World Health Organization (WHO) diagnostic criteria for stroke were used [13]. Only first-ever strokes were included. Persons with subarachnoidal haemorrhage were excluded. 

### 2.3. Interview and Measurements: All Persons in the Study

In a previous publication, definitions of stroke severity and medical risk factors have been accounted for [14]. In short, stroke severity was evaluated using the National Institutes of Health Stroke Scale (NIHSS). This scale consists of 11 items representing consciousness, vision, language, sensory, and motor function. The score ranges from 0 to 38, where 0 indicates functions within the normal range [15]. A three-item version of the Behavioural Inattention Test [16] and the Baking Tray Task [17] were used for assessment of neglect. Information on how medical risk factors and other medical conditions were diagnosed is given in Table 1.

### 2.4. Interview and Measurements: Persons Admitted to the Stroke Unit

Persons who were considered to gain most from stroke unit care were admitted to the stroke unit (SU) from the first day. Persons treated in the SU had a more comprehensive physiotherapeutic investigation. The assessments started at a median of 8 days (interquartile range 5–11) after the stroke event. If they were to be discharged before the seventh day, they were examined earlier. A structured interview was conducted in order to gather general information about each person and predictors known to be associated with falls. The persons were asked about previous falls during the preceding year. A fall was defined as an unexpected event in which a person came to rest on the ground, floor, or lower level [18]. The persons in the study self-rated their vision as normal or impaired.

### 2.5. Measurements

During the stay in the SU, the following tests were carried out: the Mini-Mental State Examination (MMSE) was used to assess cognitive function. This instrument includes six sections, orientation, registration, attention and calculation, recall, language, and copying. The score ranges from 0 to 30, and 30 points indicate normal cognitive function [19]. Birgitta Lindmark motor assessment scale (BL) was used for assessment of motor capacity. The interrater reliability for the scale is 0.96 and the intrarater reliability is 0.98 [20]. In this study we used the part that evaluates ability to perform active selective movements. The total score ranges from 0 to 57 for the upper extremity. Fifty-six points or less were considered as motor impairment. For the lower extremity the total score ranges from 0 to 36. Thirty-five points or less were considered as motor impairment. We used the Berg Balance Scale (BBS) to test balance. BBS consists of 14 items representing functional movements common in everyday life. Each task is scored on a five-point scale from 0 to 4 giving a maximum score of 56, which indicates balance ability within the normal range [21]. The inter-rater reliability and the intra-rater reliability for BBS are 0.98 and 0.97, respectively [22]. Timed Up & Go (TUG) was used as a test of basic functional mobility. The time is taken when the person rises from an armchair, walk three metres as fast as possible, cross a line on the floor, turn, walk back, and sit down again. Both inter-rater reliability and intra-rater reliability for TUG are 0.99 [23]. The results of BBS and TUG were dichotomised at points recommended in earlier studies [21, 24]. The test Stops Walking When Talking (SWWT) was used to examine whether or not a person stops walking when the examiner starts an occasional conversation [25].

### 2.6. Hip Fracture

The register at the National Board of Health and Welfare was used to identify persons with hip fracture. This register was searched for ICD-10 codes S72.0, S72.1, and S72.2, using each person's unique identification number. All persons who initially were treated in the SU and who later got a hip fracture were evaluated at the orthopaedic department, while still in hospital. In these cases, a structured interview was conducted in order to retrieve information about the fall. Persons who were not admitted to the SU at stroke onset were not interviewed when they got their hip fracture. Instead, information regarding the fall that caused their hip fracture was gathered retrospectively from their medical records.

### 2.7. Statistics

Analyses were made with purpose to compare persons with and without hip fractures. Independent sample *t*-test was used to analyse age. The Mann-Whitney test was used to analyse the scores of National Institutes of Health Stroke Scale (NIHSS) and the Mini-Mental State Examination (MMSE). The difference of proportions for dichotomised variables was calculated with Pearson Chi square test and Fisher's exact test. A *P* value of 0.05 or less was considered statistically significant. Incidence rates (*I*) were calculated according to the formula *I* = *A*/*R*, where *A* is the number of fractures during the 24-month period and *R* is the total observation time in 1000 person-years. Mortality rates (*I*) were calculated using the same formula, where *A* is the number of death during the 3-year period after stroke and *R* is the number of people in each group. Regarding fracture incidence and mortality rates confidence intervals (CI) were calculated using the Poisson distribution. The accuracy of fracture prediction is shown using sensitivity, specificity, and positive and negative predictive values.

### 2.8. Ethics

Before entering, the persons were informed that participation in this study was voluntary and that they could choose to opt out at any time without justification. They also received an information letter. If a person's ability to communicate was restricted, consent by next-of-kin was obtained. The Human Ethics Committee of the Örebro County Council and the Regional Ethics Committee in Uppsala approved the study.

## 3. Results

### 3.1. All Included Persons

377 persons with first-ever stroke (ischemic or hemorrhagic) were identified during the year of inclusion. Sixteen persons, eight women and eight men, fractured their hip within two years after stroke onset, which corresponds to an incidence of 32 (95% CI: 16–47) hip fractures per 1000 person-years. One person had two fractures within three months, one in each hip. There were no significant differences between persons with and without hip fracture, neither for the whole incidence group nor for persons treated in the SU, concerning age, gender, stroke severity, presence of neglect, or medical conditions described in Table 1.

**Figure 1:**
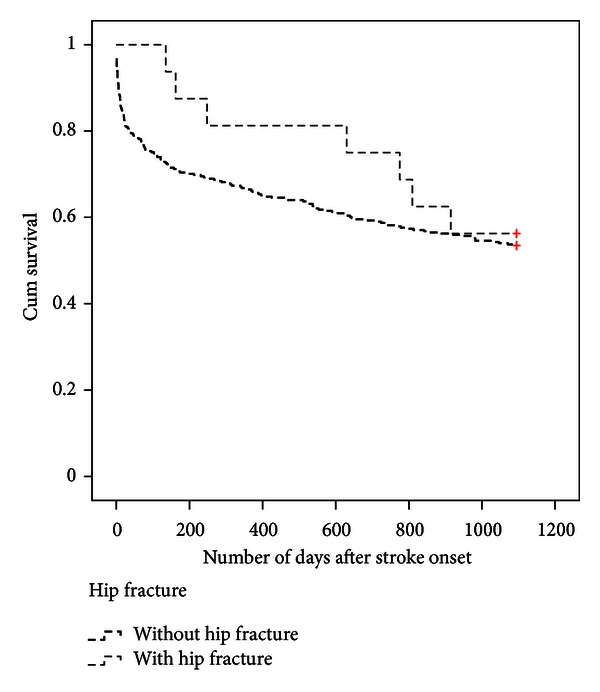
The survival time three years after stroke onset for persons with and without hip fracture.

Nine persons had the stroke lesion in the right hemisphere and seven of them in the left hemisphere. Nine persons lived in nursing homes and seven lived in their own homes. All fractures occurred after discharge from the initial hospital stay, and all persons fractured their hip indoors and in connection with a fall. Information about the circumstances when they fell and fractured their hip is given in Table 2. Eight of the sixteen persons fractured their hip within the first year and eight of them during the second year. Twelve of them fractured their hip on the stroke-affected side. Eight of the sixteen persons subsequently died during the study period, and all were dead within fifteen months after the hip fracture (4–449 days). The three-year mortality rate after stroke for persons with and without hip fracture was 44% (95% CI: 26–64) and 47% (95% CI: 42–51), respectively, (n.s. *P* = 0.827). The mortality for persons with and without hip fracture is illustrated by a Kaplan-Meier curve in Figure 1. The mean survival time for persons with hip fracture was 2.32 years (95% CI: 1.85–2.79) and for persons without hip fracture 1.91 years (95% CI: 1.78–2.04).

### 3.2. Persons Admitted to the Stroke Unit (SU)

Of the 377 persons included in the study, 218 were treated in the SU. Persons who were treated in the SU had lower mean age (75 years versus 79 years). A higher proportion of persons treated in the SU were men, and fewer had pre-stroke cognitive impairment.

Nine persons who fractured their hip were treated in the SU. When comparing the results of the functional measurements presented in Table 3, there were significant differences between persons with and without hip fracture regarding visual impairment, fractures before stroke onset, cognitive function, functional mobility (TUG), and the ability to walk and talk simultaneously (SWWT). Information about the accuracy of the fracture prediction is given in Table 4.

All persons, however, were not able to participate in all functional tests. Persons who were unable to walk were not able to participate in the test SWWT. Persons who were unable to rise from an armchair and unable to walk could not participate in TUG.

All nine persons had fallen at least once before the occasion when they fractured their hip. Five of them also had fallen before the stroke event. None of them owned or used hip protectors. 

## 4. Discussion

This study has shown a high incidence of hip fractures during two years following a first-ever stroke. Although the differences were not significant, persons who had hip fractures tended to be older and to have lower stroke severity. More persons with hip fractures who were treated in the stroke unit had impaired vision. More of them had had fractures before stroke onset, and they had lower cognitive function. The results of Timed Up & Go and Stops Walking When Talking differed between persons with and without hip fracture. All persons sustained their hip fracture indoors, after the initial hospital stay, while performing everyday activities.

MMSE was significantly lower in persons with hip fractures compared with persons without hip fracture, and there was also a trend for persons with fracture to more often have cognitive impairment prior to the stroke event. Cognitive impairment is a predictor for falls [26], and nearly half of persons with a diagnosis of hip fracture have dementia [27]. Persons with cognitive impairment may have reduced ability to make adequate decisions of their capability to perform everyday activities, which may lead to a higher level of risk taking.

Given the wide confidence interval, the fracture incidence of 32 per 1000 person-years must be treated with caution. It is higher than 7 per 1000 person-years which Dennis et al. [9] have reported but may possibly be comparable to 17 per 1000 person-years and 19.8 per 1000 person-years as reported by Ramnemark et al. [5] and Kanis et al. [3], respectively. What might possibly explain the higher incidence in our study is that due to the community-based design, more persons with cognitive impairment may have been included in our study, thus increasing the incidence of falls and fractures. 

Persons who had hip fractures tended to have milder strokes. An explanation may be that persons with severe strokes are less mobile and therefore not able to expose themselves to the risk of falling. This is also the most likely reason for persons without hip fracture to have a shorter survival time. A large proportion of persons in both groups had impaired balance, but our study failed to show that BBS predicted hip fractures. Therefore, it seems clear that it is not stroke severity nor impaired balance *per se* that is the most important predictor of fractures. This does not mean that they are irrelevant, but the ability to adapt to these impairments may be more important. Our study also showed that visual impairment is an important predictor for falls and hip fracture in older people. This is consistent with a previous study [28].

In the present study, where only persons with first-ever strokes were included, there was a significant difference regarding TUG and SWWT between persons who later on fractured their hip and those who did not. Previous studies have shown that both TUG [29] and SWWT [25, 30] are able to predict falls and fractures in persons with different medical conditions. Also it has been shown that persons with stroke have significantly longer TUG time than age-matched controls, and it takes significantly longer time for them to accomplish the turning moment in TUG [31]. In their original study, Lundin-Olsson et al. found that an even higher proportion of the persons was positive in SWWT than in our study [25], but in that study a larger proportion of the participants suffered from cognitive impairment. Our interpretation is that the results of TUG and SWWT demonstrate that persons with impaired cognition have difficulties in performing a complex series of activities and that they show a reduced capacity for multitasking.

The accuracy of the fracture prediction differed as shown in Table 4. In general (with exception for TUG), the specificity was higher than the sensitivity, and the negative predictive value was higher than the positive predictive value. That means that TUG may be better at identifying persons at risk for fracturing their hip and that the other tests may be better at identifying persons that are not.

A strength of this study is that it is community based, which means a minimum of selection bias. The case ascertainment regarding hip fractures should be complete, because all cases of hip fractures are hospitalized and therefore are registered in the person register of the National Board of Health and Welfare, as well as in the local person administration system. A limitation of this study is that not all persons were treated in the SU and therefore not fully evaluated by a physiotherapist. We also lack complete information about the drug use, especially by persons who were not treated in the SU. A further limitation is that we relied on self-rating of vision and self-reporting when documenting previous fractures and falls. Recall bias may have played a role. Erroneous coding of fractures, or treatment of hip fractures abroad, may have led to having a few hip fractures missed.

## 5. Conclusions

Hip fractures after stroke is common, even more common than previously described. Perhaps unexpectedly, high stroke severity is not associated with fractures. Based on observations on persons who were treated in the stroke unit, visual disturbance and cognitive impairment do seem to play important roles. The fractures occurred indoors, in the person's homes, in everyday situations. This leads us to conclude that ambulatory persons with previous fractures, with cognitive or visual impairment, are at particular risk. Such persons should be targets for fall preventive measures, particularly directed to the situation in the person's own home. In addition to cognitive and visual tests, TUG may be used to identify persons with an increased risk for fractures.

## Figures and Tables

**Table 1:** Diagnosis of associated medical conditions.

Prestroke dementia	Assessment was based on interviews with the persons and a knowledgeable informant. We required the presence of memory impairment as well as deficits in other cognitive abilities according to the ICD-10 classification

Atrial fibrillation (AF)	A documented history of paroxysmal AF, alternatively if AF was present at hospital admission

Heart failure and myocardial infarction	Documentation of these conditions in medical records

Hypertension	When the presence of hypertension was documented in medical records, alternatively if ≥2 readings of systolic blood pressure were ≥160 mm Hg, or diastolic blood pressure was ≥95 mm Hg at any time before the onset of stroke

Pre-stroke transient ischemic attack (TIA)	Documentation of TIA in medical records or from the person's own report

Diabetes mellitus	If a person gave a history of diabetes mellitus that was confirmed by their medical records or was taking insulin or an oral hypoglycemic agent

Epilepsy	Epilepsy was diagnosed from the hospital's patient register (diagnosis code G40) and confirmed from the patient records. Epilepsy could be present both before as well as at any time after the stroke

**Table 2:** The circumstances that prevailed when the persons sustained the hip fracture.

		NIHSS	MMSE
Man, 84 years	He fell when he put his glasses on the small table beside his bed	5	27
Man, 86 years	He fell on his way from the bedroom to the kitchen	2	23
Woman, 73 years	She fell when the door of the elevator hit her	6	29
Man, 77 years	The staff at his nursing home found him on the floor in the bathroom	2	20
Woman, 84 years	She lost the grip and fell when she tried to draw herself up from the wheelchair	6	21
Woman, 94 years	She fell backwards into the wheelchair but it turned over and she fell on the floor	12	15
Man, 89 years	He fell on his way from the bedroom to the kitchen	0	13
Man, 58 years	On his way from the bedroom to the front door, he stumbled on a carpet and fell	2	24
Man, 87 years	He was going to sit down but missed the chair and fell on the floor	8	12
Man, 69 years	He was going to sit down in his wheelchair but missed it and fell on the floor	4	—
Woman, 87 years	During the night she fell in her home	2	—
Man, 81 years	He arrived at the hospital after fall in her home	5	—
Woman, 76 years	She fell in her home and hit her hip	8	—
Woman, 86 years	She fell from a chair	3	—
Woman, 83 years	She fell during the evening and hit her hip	2	—
Woman, 86 years	Unspecified fall. She complained from pain in the right leg	7	—

The scores of the National Institutes of Health Stroke Scale (NIHSS) and the Mini-Mental State Examination (MMSE). The last seven persons were not initially treated in the stroke unit. For these persons MMSE was not performed.

**Table 3:** Characteristics of persons admitted to the stroke unit (*n* = 218) with hip fracture (H) and without hip fracture (NH).

	Persons with hip fracture (*n* = 9)	*P*	Persons without hip fracture (*n* = 209)	*n* tested	
				H	NH
Age, mean (range) years	80 (56–92)	0.18	75 (33–94)	9	209
Men, *n* (%)	6 (67)	0.50	105 (50)	9	209
NIHSS, md (IQR)	5 (2–7)	0.18	6 (3–11)	9	209
Neglect, *n* (%)	2 (22)	0.96	45 (22)	9	209
Visual impairment, *n* (%)	6 (67)	0.04	52 (33)	9	156
Persons who fell during the previous year, *n* (%)	5 (56)	0.76	59 (39)	9	156
Persons who fell at the stroke unit, *n* (%)	1 (11)	0.84	17 (9)	9	187
Persons with fractures before stroke onset, *n* (%)	2 (22)	0.01	6 (4)	9	156
MMSE, md (IQR)	21 (14–26)	0.05	26 (21–28)	9	152
BL: motor impairment upper ex, *n* (%)	7 (88)	0.18	102 (64)	8	159
BL: motor impairment lower ex, *n* (%)	5 (63)	1	99 (62)	8	159
BBS < 45, *n* (%)	6 (75)	0.28	77 (50)	8	154
TUG ≥ 14 seconds, *n* (%)	4 (80)	0.05	37 (34)	5	110
SWWT, *n* (%)	2 (29)	0.02	7 (6)	7	118

NIHSS: National Institutes of Health Stroke Scale; Md: median; IQR: interquartile range; MMSE: Mini-Mental State Examination BL: Birgitta Lindmark motor assessment scale; BBS: Berg Balance Scale; TUG: Timed Up & Go; SWWT: Stops Walking When Talking.

**Table 4:** Accuracy of the fracture prediction.

	Prevalence of hip fracture	Sensitivity % (*n*)	Specificity % (*n*)	Positive predictive value % (*n*)	Negative predictive value % (*n*)
Impaired vision	5 (9/165)	67 (6/9)	67 (104/156)	10 (6/58)	97 (104/107)
Previous fractures	5 (9/165)	22 (2/9)	96 (150/156)	25 (2/8)	96 (150/157)
TUG ≥ 14 s	4 (5/115)	80 (4/5)	66 (73/110)	10 (4/41)	99 (73/74)
SWWT	6 (7/125)	29 (2/7)	94 (111/118)	22 (2/9)	96 (111/116)

TUG: Timed Up & Go; SWWT: Stops Walking When Talking.
